# Assessing cardiovascular disease risk factor screening inequalities in India using Lot Quality Assurance Sampling

**DOI:** 10.1186/s12913-020-05914-y

**Published:** 2020-11-25

**Authors:** Devaki Nambiar, Soumyadeep Bhaumik, Anita Pal, Rajani Ved

**Affiliations:** 1 George Institute for Global Health, 311-312, Third Floor, Elegance Tower, Plot No. 8, Jasola District Centre, New Delhi, 110025 India; 2 Faculty of Medicine, University of New South Wales, Sydney, Australia; 3 Prasanna School of Public Health, Manipal Academy of Higher Education, Manipal, India; 4 Bernard Lown Scholars for Cardiovascular Health Program, Harvard T. H. Chan School of Public Health, Boston, USA; 5 Department of Education and Education Technology, University of Hyderabad, Hyderabad, India; 6 National Health Systems Resource Centre, New Delhi, India

**Keywords:** Lot quality assurance sampling, Cardiovascular diseases, Risk factors, Socioeconomic factors, Mass screening, India, Primary health care

## Abstract

**Background:**

Cardiovascular diseases (CVDs) are the leading cause of mortality in India. India has rolled out Comprehensive Primary Health Care (CPHC) reforms including population based screening for hypertension and diabetes, facilitated by frontline health workers. Our study assessed blood pressure and blood sugar coverage achieved by frontline workers using Lot Quality Assurance Sampling (LQAS).

**Methods:**

LQAS Supervision Areas were defined as catchments covered by frontline workers in primary health centres in two districts each of Uttar Pradesh and Delhi. In each Area, 19 households for each of four sampling universes (males, females, Above Poverty Line (APL) and Below Poverty Line (BPL)) were visited using probability proportional to size sampling. Following written informed consent procedures, a short questionnaire was administered to individuals aged 30 or older using tablets related to screening for diabetes and hypertension. Using the LQAS hand tally method, coverage across Supervision Areas was determined.

**Results:**

A sample of 2052 individuals was surveyed, median ages ranging from 42 to 45 years. Caste affiliation, education levels, and occupation varied by location; the sample was largely married and Hindu. Awareness of and interaction with frontline health workers was reported in Uttar Pradesh and mixed in Delhi. Greater coverage of CVD risk factor screening (especially blood pressure) was seen among females, as compared to males. No clear pattern of inequality was seen by poverty status; some SAs did not have adequate BPL samples. Overall, blood pressure and blood sugar screening coverage by frontline health workers fell short of targeted coverage levels at the aggregate level, but in all sites, at least one area was crossing this threshold level.

**Conclusion:**

CVD screening coverage levels at this early stage are low. More emphasis may be needed on reaching males. Sex and poverty related inequalities must be addressed by more closely studying the local context and models of service delivery where the threshold of screening is being met. LQAS is a pragmatic method for measuring program inequalities, in resource-constrained settings, although possibly not for spatially segregated population sub-groups.

**Supplementary Information:**

The online version contains supplementary material available at 10.1186/s12913-020-05914-y.

## Background

Cardiovascular diseases (CVDs) are the leading cause of mortality in India [1, 2]. It has been projected that India will lose 16 million potentially productive years of life due to premature cardiovascular deaths among those aged 35–64 over the next 34 years [3]. CVDs additionally contribute to escalating health expenditure both within the healthcare system and by individuals [4–6], particularly in the case of multiple morbidities [7, 8]. CVD mortality, morbidity, and risk factors are known to affect equity groups variably: those with lower socio-economic status (i.e. lower incomes and education) are more vulnerable than those with higher socio-economic status [9–12]. While some evidence suggests that acute coronary outcomes may not vary significantly by sex in India [13], a greater burden of risk factor among males has been observed globally and in Indian studies as well [14–16].

Given the growing burden of CVDs nationwide [11, 17, 18], and the increasing push towards Universal Health Coverage (UHC), India has been seeking to redesign the scope of health care delivery across the spectrum [19–21]. In 2016, the Government of India launched policies and guidelines to support the rollout of Comprehensive Primary Health Care including prevention and control of Non-Communicable Diseases (CPHC-NCD) [21]. The programme envisions a key role for Auxiliary Nurse Midwives (ANMs) and community health workers (called Accredited Social Health Activitists (ASHAs)) - in CVD prevention and control- thus envisaging a more horizontal and diverse role for them [21, 22].

ASHAs are required to enumerate populations, implement a risk profile checklist, motivate those over 30 years for screening and prevention of hypertension and diabetes (this is considered the ‘eligible population’ for screening, and for our study), promote cessation of tobacco use and lifestyle modification, refer the high-risk for further diagnosis and treatment, and monitor uptake of services to minimize exclusion [22].

CPHC-NCD services were being slowly being rolled out in different states in the period 2017 onwards. Monitoring indicators, related to screening, treatment and follow-up service coverage were also introduced soon after the program: they include an indicator on proportion of those eligible (i.e. aged 30 years or over) whose blood pressure was measured in the previous 2 years, as well as the proportions of those screening positive examined at the primary public health centre, those with initiated treatment remaining on treatment, and those on treatment with conditions under control [22]. At the outset of the program and in training, heavy thrust was placed on enumeration of families and on promotion of blood pressure and blood glucose screening. As part of the guidelines, it was envisioned that screening coverage of 50% of the eligible population could be achieved in the first year, 65% in the second year and 80% thereafter [22]. In order to achieve these coverage rates, ASHAs were to expand their scope of work from maternal and child health to families, with an emphasis on those aged 30 and older as part of this program [23]. This would involve at least one visit to enumerate families, administer a Community Based Assessment Checklist and motivate all eligible persons, especially those at higher risk for screening – although no specific guidance on this is currently in place. Rather, it was proposed that frontline workers receive incentive payments for the number of families enumerated and the number of individuals getting screening in the year.

Existing evidence from India suggests that frontline health workers are ‘unrecognised’ and ‘overburdened’ members of NCD service delivery teams [24], but also that in trial settings, they can retain increase treatment-seeking among high risk individuals [25], and even reductions in some risk factors, like hypertension [26].

We didn’t find studies on how impacts are distributed across populations subgroups, however. At the systems level, moreover, global evidence on the inverse equity hypothesis (where those with advantage disproportionately benefit from new programs causing inequality) [27], and the existing evidence of socio-economic inequality in the early rollout of MCH -focused services [28], implementers were concerned about inequality. Operations research was needed to understand how service delivery under CPHC-NCD was progressing, particularly for underserved groups. This was seen to be important given the sex-related differences in CVD risk profiles seen globally and in India [9, 13, 29, 30] as well as differences in health service utilisation by gender and socio-economic status [31–33], although the latter is focused on inpatient care-seeking. Disaggregated evidence on CVD service coverage at the primary level in India is less common, but all the more needed, given the recent rollout of this scheme at the primary care level.

Understanding service coverage using an inequality lens is challenging, as disaggregated data for this purpose typically requires a large sample size. An exception is offered by the method of Lot Quality Assurance Sampling (LQAS), which allows the use of relatively smaller sample sizes to assess differences in screening with the possibility of providing immediate programmatic feedback to health system actors [34]. We employed this method to meet our study goal of assessing blood pressure and blood sugar screening coverage by sex and poverty status in two Indian states.

## Methods

### Study design

The LQAS method uses stratified random sampling to assess whether coverage/quality in a management stratum (called supervisory area or SA) exceeds a specific performance threshold. It is essentially a classification and quality control system originating in industrial production where batches or lots of products manufactured in a factory were classified as ‘high’ or ‘low’ [34]. The dichotomous classification system was created to enable valid and effective monitoring with small sample sizes.

LQAS has been adapted for use in public health in various contexts [35] and has also been integrated for monitoring and evaluation for large programs [36, 37] to monitor coverage of maternal, neonatal and child health services – particularly immunisation – as well as communicable disease-related data collection in Uganda, South Sudan, Benin, Uzbekistan, Nigeria, and Turkey [37–43]. Applications in India include monitoring of measles immunisation in Tamil Nadu and malaria treatment management in Odisha [36, 44, 45]. LQAS methodology in public health is designed to allow local program managers to dichotomously classify whether local administrative units, also called lots or Supervision Areas have reached health service or program targets (threshold) without specifically measuring the coverage for the entire population in that area. This helps pinpoint which areas need targeted service coverage improvement (i.e. those below the threshold) and which can offer lessons on how improvements could be brought about (i.e. those above the threshold). Pooling of lots into the larger administrative areas allows sufficient sample size of the coverage at the larger administrative area without knowing the specific coverage of the local administrative units.

In our application of this method, we created lots or Supervision Areas to assess coverage and inequalities in screening, for hypertension and diabetes achieved by frontline health workers in two Indian states in the 2 years following the rollout of the CPHC-NCD program. We wanted to enable local use and application of data, while not placing a high burden on data collection. This is the first instance, to our knowledge, of the use of this methodology to examine quality of NCD data using an inequality-focused approach anywhere.

### Sampling

**Fig. 1:**
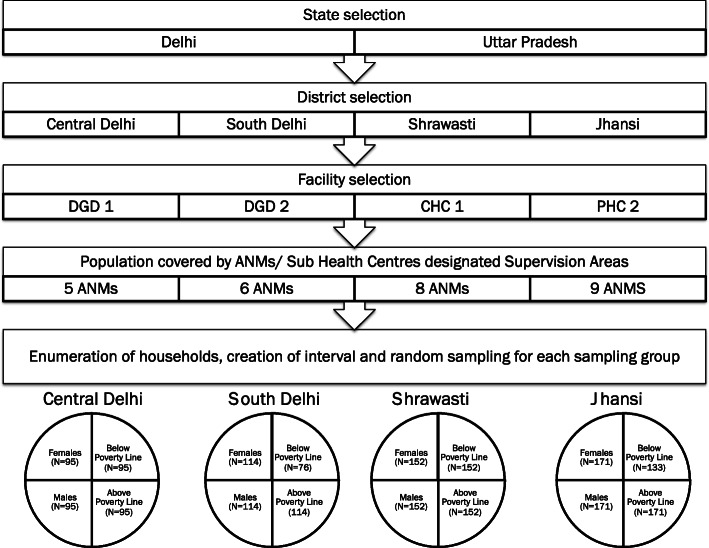
Sampling design. Note: *DGD* - Delhi Government Dispensary; *CHC* - Community Health Centre; *PHC* - Primary Health Centre; *ANM* - Auxiliary Nurse Midwife

State and district selection: The two states selected for this study were Delhi and Uttar Pradesh (see Fig. 1). They were purposively chosen because a) they represent diverse habitational typologies (Delhi is largely urban while Uttar Pradesh is largely rural); b) recent data suggests a substantial and growing burden of diabetes and hypertension in these states; and c) their respective health systems are transitioning from MCH-driven care to CPHC, including NCD prevention and control.

In each state, districts were chosen based on three criteria. First, median ranking on the National Health Mission’s Health Management Information System (HMIS) Composite Index score of care for reproductive health, pregnancy care, child birth and newborn care. The assumption here is that those districts with close to median MCH readiness should be in a position to scale up the CPHC-NCD programme with reasonable success. Second, these districts were the ones where there was a prior track record of health systems and/or CVD-related research by the National Health Systems Resource Centre, thus extending our existing partnership with states. Districts shortlisted under these criteria were referred to state officials for their concurrence for finalization.

Selection of facilities and supervision areas: Within selected districts, HMIS data were reviewed, triangulated, with data from the District Level Health and Facility Survey (2012–13) and the National Family Health Survey (the NFHS, India’s Demographic and Health Survey (2015–2016). PHCs were ranked by human resource availability in consonance with the Indian Public Health Standards for NCD service provision. Given that the CPHC programme was just being rolled out, consultations were held in both states to ensure that the CPHC-NCD programme rollout is already underway in facilities shortlisted and chosen.

In our surveys, the catchment area of the ANM served as a Supervision Area because this would be the most conducive to generating local learning at the facility level, course correction involving the primary health care structure, and impact at the community level. In Uttar Pradesh’s Shrawasti district, areas served by 8 ANMs were chosen, while in Jhansi, 9 were identified. In Delhi, five Supervision Areas were chosen in the Central district while 6 were chosen in the South. In each area, for each population of interest (i.e. male/female/above poverty line/below poverty line aged 30 years or more), LQAS requires a sample size of 19 so that α and β errors do not exceed 10% [46].

Sampling of households and eligible participants: A staged sampling design was used. In the first stage, probability proportional to size sampling was used to identify 19 interview locations (this was villages in rural UP and ward subplots in urban Delhi) in each Supervision Area. A list of all the communities in each Supervision Area and their population sizes was obtained from the respective State Health Missions for this purpose. For each Supervision Area, its cumulative population was calculated, and the sampling interval was found to divide the total population by sample size (19 per Supervision Area). A random number, using a random number table, was added to the sampling interval to identify the number of interviews required in each location (19 interview locations per sampling group – i.e. females, males, BPL, APL – per Supervision Area).

In the next stage, for each interview location, segmentation sampling was done to randomly select a household using standard LQAS methods. This process was carried out by a team comprising three data collectors. One of the data collectors would draw up a map with support from a community leader or local health worker. Following this, using a walking rule devised for both rural and urban contexts, the remaining two data collectors would carry out sampling of households, defined as a dwelling within which a group of people eat together from the same cooking stove, consistent with the Indian Census [47].

In the final phase, a randomly selected eligible respondent (i.e. aged 30 years or above, as per the national guidelines) in each sampled household was interviewed. In order to be able to determine screening coverage by socio-economic status and sex, we created four LQAS sampling groups: male, female, Above the Poverty Line (APL), and Below the Poverty Line (BPL).^1^ In each Supervision Area, one data collector was responsible for sampling 19 female and 19 males while the other, 19 BPL and 19 APL individuals – each from separate households (i.e. no more than one person per household was sampled in any area overall). Sex was determined by observation and poverty status confirmed by reviewing, with permission, government issued ration cards (during piloting we determined the vernacular used to describe these cards and the colour coding based on which APL and BPL status was indicated in each state).

### Data collection, entry and analysis

#### Tools

A 31 item questionnaire for this study was developed by the research team at TGI in English and Hindi, and piloted in both states (See Supplemental File 1). Basic socio-demographic characteristics were included, alongside indicators (whose performance targets to determine ‘d’ were developed) based on the M&E framework for the CPHC NCD programme across phases of awareness-raising, screening, treatment and follow-up, in consultation with experts from the National Health Systems Resource Centre, tasked with providing guidance for program rollout.

#### Field procedure

Following the sample selection outlined above, individuals were approached for interviews. Written informed consent was obtained after explaining the purpose of the study to each participant and sharing bilingual participant information sheets. Data was gathered between October of 2018 and January of 2019.

#### Data management and analysis

Data was exported into a Structured Query Language (SQL) database. A checklist was created to assess output on key indicators (such as ANM code, sampling group, written informed consent, respondent’s age, gender and BPL card holder), and reviewed frequently throughout process of fieldwork. Supervisors were regularly provided with sampling adjustment sheets to help identify certain errors that may have occurred during the course of completing interviews. The use of an app also enabled us to run extensive quality checks and data inconsistencies, which helped improve quality of the data.

All data collected through survey was exported into a simple excel spreadsheet and then imported into Microsoft Excel and STATA 13 for analysis. Basic descriptive analyses were generated in keeping with the requirements of LQAS analysis – using hand tallies in Microsoft Excel. Threshold levels do not exist for knowing or interacting with an ASHA in India, although it is expected that they will have very high coverage. We set a threshold of 90% for knowing the area ASHA. With regard to having interacted in the past year, we pegged the threshold at 80%, the highest coverage level expected in the NCD-CPHC program mindful of the fact that these workers would be approaching households in the area for MCH and enumeration activities in the course of the past year, and approximating an 85.9% screening coverage achieved in a study underway contemporaneously [25]. Thresholds for the NCD screening indicators were decided a priori based on the guidelines, which suggested at least 50% screening coverage in year one and 65% in year two. The target thresholds for each indicator are mentioned in each table (see Tables 2, 3 and 4).

### Ethical considerations

Ethics approval was obtained from the Institutional Ethics Committee of The George Institute for Global Health, India (TGI). The data collected was protected from unauthorized access and was available only to researchers involved in this study, stored on local servers. To ensure this, data was be encoded and saved on encrypted and password protected computers/drives that have specifically been procured for this study. Secondary data analysis was undertaken with large datasets that are unlinked and de-identified. Any identifying information was used for recruitment purposes only.

## Results

### Socio-demographic profile of participants

Table 1 indicates the socio-demographic profile of participants in our study. In each of the four districts of the study, a higher proportion of females as compared to males was sampled overall. Median age ranged from 41 to 45 years across sites. The percentage of participants who were illiterate or with education up to or below primary level were 89.7 and 65.5% respectively across the two mostly rural locations of Shrawasti and Jhansi, with 66.1 and 42.1% in the two mostly urban locations of Central and South Delhi; 33.5% participants in South Delhi were enrolled in or had higher than secondary school education. A majority of participants were Hindu, while in Shrawasti, 16.45% of participants were Muslims. In Jhansi and Shrawasti, a higher percentage of participants were from Other Backward Classes, while in Central and South Delhi, Scheduled Caste and General groups were most represented, respectively. Most participants across all the locations were married, although 15.3% of participants in Central Delhi reported being widow (er)ed. The proportion of participants who were homemakers in Central and South Delhi was higher at 58 and 54%, respectively, compared to Shrawasti and Jhansi, where self-employment in agriculture was commonly reported.

**Table 1:** Socio-demographic profile of participants

Background characteristics		Number (proportion)			
		Central Delhi	South Delhi	Shrawasti	Jhansi
Total Sample Size		380	418	608	646
Gender	Female	249 (65.5)	244 (58.4)	335 (55.1)	388 (60.1)
	Male	131 (34.5)	174 (41.6)	273 (44.9)	258 (39.9)
Age	Median	45	42	44	45
	Range	30–85	30–79	30–90	30–95
Level of education	Illiterate	169 (44.5)	115 (27.5)	493 (81.1)	295 (45.7)
	Up to primary or below	82 (21.6)	61 (14.6)	52 (8.6)	128 (19.8)
	Primary to secondary	74 (19.5)	102 (24.4)	32 (5.3)	119 (18.4)
	Higher than secondary	55 (14.5)	140 (33.5)	31 (5.1)	104 (16.1)
Religion	Hindu	353 (92.9)	390 (93.3)	508 (83.6)	639 (98.9)
	Muslim	25 (6.6)	22 (5.3)	100 (16.5)	7 (1.1)
	Christian	_	6 (1.4)	_	_
	Don’t know/won’t say	2 (0.5)	_	_	_
Caste	General	89 (23.4)	171 (40.9)	65 (10.7)	84 (13.0)
	Other Backward Classes	121 (31.8)	125 (29.9)	374 (61.5)	317 (49.07)
	Scheduled Caste	160 (42.1)	100 (23.9)	154 (25.3)	199 (30.8)
	Scheduled Tribe	10 (2.6)	15 (3.6)	15 (2.5)	44 (7.0)
	Don’t know	_	7 (1.7)	_	1 (0.2)
Marital Status	Never	2 (0.5)	4 (0.9)	4 (0.7)	6 (0.9)
	Married	319 (84.0)	387 (92.6)	571 (93.9)	579 (89.6)
	Separated/divorced	1 (0.3)	_	1 (0.2)	1 (0.2)
	Widowed	58 (15.3)	27 (6.5)	32 (5.3)	60 (9.3)
Profession/ occupation	Unemployed	10 (2.6)	19 (4.6)	17 (2.8)	35 (5.4)
	Agriculture-self employed	_	_	189 (31.1)	140 (21.7)
	Agriculture-labour	8 (2.1)	2 (0.5)	142 (23.4)	108 (16.7)
	Non-agriculture labour/casual labour	20 (5.3)	21 (5.0)	20 (3.3)	39 (6.04)
	Non-agriculture-self employed	58 (15.3)	51 (12.2)	15 (2.5)	11 (1.7)
	Service/salary	64 (16.8)	97 (23.2)	11 (1.8)	47 (7.3)
	Home maker	220 (57.9)	228 (54.6)	212 (34.9)	266 (41.2)
	Other	_	_	2 (0.3)	_

### Performance of supervision areas in CVD risk factor screening coverage in females and males

We observed in some Supervision Areas in both Delhi and Uttar Pradesh that coverage of blood pressure and blood sugar screening was below the threshold in both sexes. In Central Delhi, sex differences were apparent (see Table 2). We noted that in some Supervision Areas (1, 3, 4, and 5), males did not reach the threshold for any screening indicator, whereas for females, knowing the area ASHA and blood pressure screening coverage was achieved. In Supervision Area 2, males and females did not meet the threshold for knowing and meeting their area frontline worker, but both sexes met the threshold for blood pressure screening in the past year. In South Delhi, females, and not males, more often achieved the target coverage for knowing and interacting with frontline workers. In three Supervision Areas, females also appeared to achieve the target for blood pressure screening, while males did not. In Supervision Areas 2 and 5, we found that women achieved target coverage across all indicators, while the same was not true for men. In Supervision Area 4, women did not achieve screening coverage, while men did. In Supervision Areas 1–3, and 5, 7, and 8 of Shrawasti, blood pressure screening met target coverage among females only. In Area 8, even blood sugar screening exceeded 50% of the female population. In Jhansi, we noted that sex related differences in knowing frontline workers were not there and neither sex achieved coverage thresholds in five of nine Areas. In Supervision Areas 1 and 6, both sexes had achieved the target coverage for blood pressure measurement in the last year.

**Table 2:**
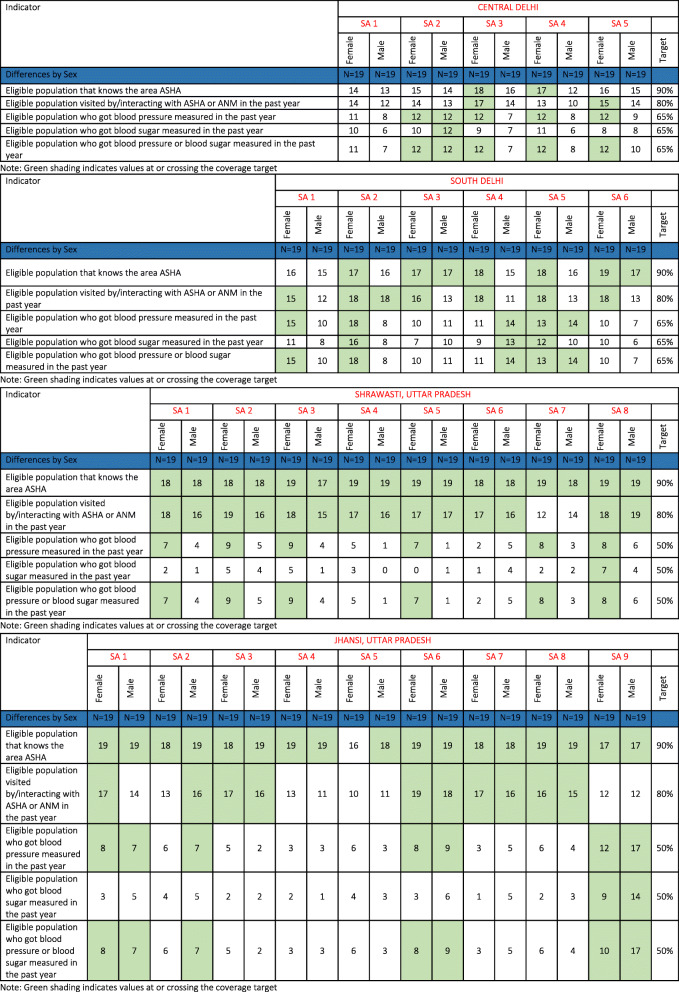
Frontline worker and ncd screening coverage disaggregated by sex

Note: Green shading indicates values at or crossing the coverage target

### Performance of supervision areas in CVD risk factor screening coverage in BPL and APL persons

We were unable to identify BPL populations in four of our Supervision Areas (1 and 2 in South Delhi and 4 and 9 in Jhansi), despite carrying out extensive attempts at sampling (they are therefore excluded from all analyses). This may be attributable to the housing typologies in these areas, which were associated with wealth (i.e. these were wealthy residential areas). We were thus unable to make inferences about poverty-related coverage differences in these Supervision Areas.

In Central Delhi, there were mixed patterns of screening coverage (see Table 3). For one, in only one Area did the APL population cross the threshold for knowing the area ASHA. We found two Areas (3 and 5) where BPL, but not APL samples achieved the blood pressure screening threshold, while in another two (1 and 2), both did. In South Delhi, two of the Supervision Areas did not have an adequate BPL sample for us to determine difference, as aforementioned. We noted that in the APL sample, all coverage targets were met in Supervision Area 1. Further, Supervision Area 5 met all coverage targets, with no differences by poverty status, while in Area 3 BP coverage was achieved. Finally in Supervision Area 6, only the APL sample achieved coverage. In Supervision Areas 1 and 3 of Shrawasti, coverage of APL populations was crossing the threshold while this was not the case of BPL. In Supervision Area 7, however, the BP screening coverage proportions targets were met for BPL populations but not for APL. In the case of Supervision Areas 4–6, screening levels were below the threshold for all groups. Supervision Area 1 of Jhansi achieved blood pressure coverage across populations while Areas 2 and 8 met targets in APL but not BPL groups. Supervision Areas 3–7 had no population achieving target screening coverage while 9 (without a BPL sample) met coverage levels in APL persons.

**Table 3:**
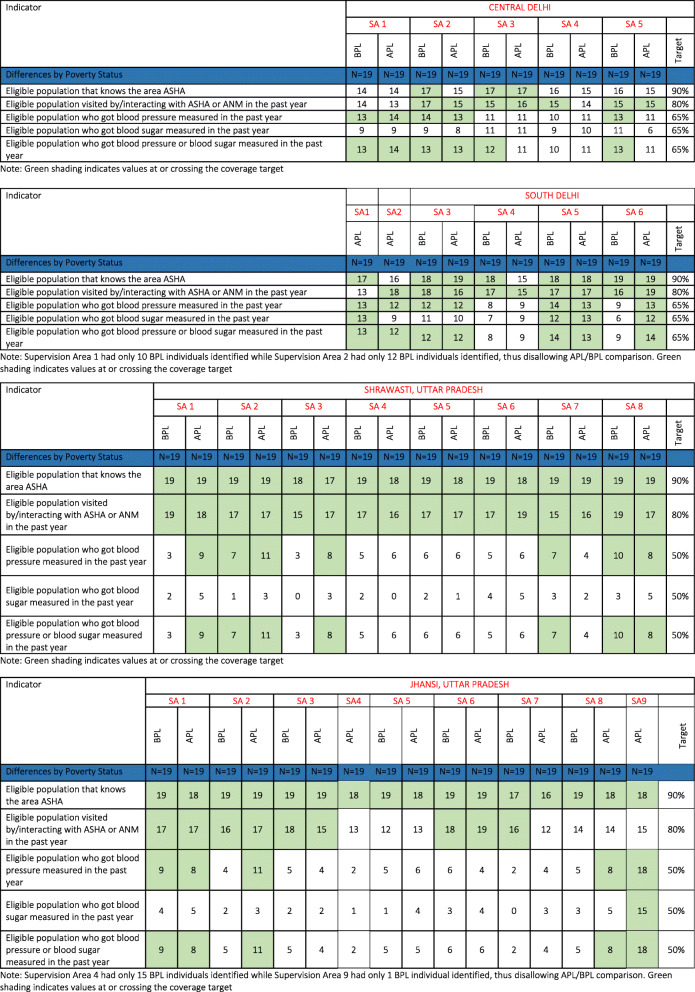
Frontline worker and ncd screening coverage disaggregated by poverty status

Note: Green shading indicates values at or crossing the coverage target

Note: In South Delhi, Supervision Area 1 had only 10 BPL individuals identified while Supervision Area 2 had only 12 BPL individuals identified, thus disallowing APL/BPL comparison. Green shading indicates values at or crossing the coverage target

Note: In Jhansi, Supervision Area 4 had only 15 BPL individuals identified while Supervision Area 9 had only 1 BPL individual identified, thus disallowing APL/BPL comparison. Green shading indicates values at or crossing the coverage target

### Overall performance for key NCD coverage indicators

Average blood pressure or blood sugar screening coverage rates in both Delhi (58.9% in Central and 61.5% in South, both less than 65%) and Uttar Pradesh (30.9% in Shrawasti and 33.4% in Jhansi, both less than 50%) were below the set thresholds (see Table 4). However, in all districts, there was at least one Supervision Area that was crossing the threshold. For instance, at least half of Supervision Areas in both Delhi sites crossed the threshold for BP measurement in the past year. Even in Shrawasti, where the proportion of those screened for blood sugar in the past year was just 13.7%, one Supervision Area did have coverage of 50% or more. These variations are not typically visible in aggregate measures.

**Table 4:**
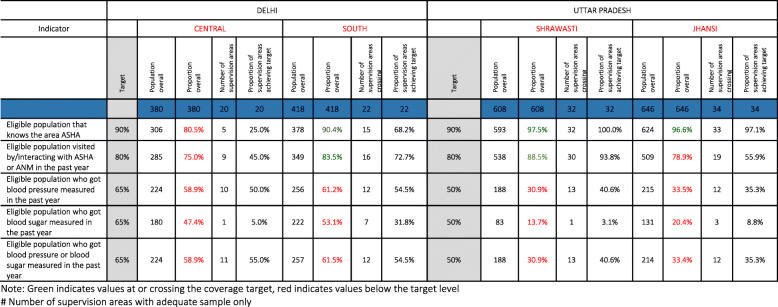
Frontline worker and ncd screening coverage in overall population and supervision areas

Note: Green indicates values at or crossing the coverage target, red indicates values below the target level

# Number of Supervision Areas with adequate sample only

## Discussion

This study sought to identify whether areas in Delhi and Uttar Pradesh met threshold levels of blood pressure and blood pressure screening coverage among females, males, APL and BPL populations. NCD screening coverage favoured women generally (and particularly in Shrawasti), possibly because existing RMNCAH (Reproductive, Maternal, Newborn, Child and Adolescent Health) services offer critical entry points to screen women for NCDs. In Uttar Pradesh, there is still a major thrust on RMNCAH care provision, including blood pressure management and more recently, management of gestational diabetes. Women with high blood pressure in pregnancy are up to four times more likely to develop long-term high blood pressure and women who have diabetes in pregnancy are seven times more likely to develop type 2 diabetes [52]. Thus, intervention during pregnancy could be a great gateway to promote health through the life-course [53]. What was striking, however, was that a large proportion had gotten their blood pressure measured in the past year, and since the age of participants was older than the typical age span of reproduction, RMNCAH services alone may not explain this difference. Furthermore, such is not the case with blood sugar measurement, which was on the whole reported by fewer persons across sexes.

While we were not able to find studies on coverage of CVD risk factor screening per se in India, multi-sited studies from India have found higher CVD risk factor prevalence – including diabetes and hypertension – among males as compared to females [15, 16, 30]. This being the case, our finding, that males are less likely to be screened in these initial days of program rollout, suggests that emphasis needs to be placed on reaching males.

Our study revealed areas where there were inadequate samples of BPL populations to determine coverage for this group. Where sample was adequate, we found no consistent patterns in screening coverage by poverty status, suggesting that local context has strong bearing on access. In some Supervision Areas, screening favoured APL, but we also found Supervision Areas with neither group achieving coverage. One Supervision Area in Jhansi achieved threshold levels by both sex and poverty status, but did not have a BPL sample. It is possible that this was an area with higher socio-economic status; further study is required to determine whether this was part of the reason why screening access was so high. Evidence in India and globally suggests that wealthier income groups have greater access to screening services, and rely more on the private sector [54, 55]. Our data also revealed that in Shrawasti overall, even though 9% of BPL persons reported being diagnosed with hypertension or diabetes in the past year, none were on treatment. This is an area that we intend to explore further.

A pooled study of NCD prevalence in LMICs found inverse associations between socio-economic status and prevalence of angina, asthma, arthritis and depression to the detriment of lower socioeconomic groups [56]. A global meta-analysis found that lowest levels of socio-economic status were associated with greater risk of CVD in men and women; authors posit that “an additional explanation for the observed sex differences observed for [Coronary Heart Disease] CHD and CVD may be differential identification of high CHD and CVD risk and access and adherence to preventative treatment and risk factor management for men and women across levels of socioeconomic disadvantage” [14]. Our research did not yield clear patterns by socio-economic status, which could be related to our operationalisation of socio-economic status. APL and BPL were two sampling groups where quintiles or other groupings would have increased our sample size but perhaps yielded different results.

As suggested by the aforementioned studies, future research and program monitoring should be directed towards understanding how screening coverage is among persons with high CVD risk– across sexes, socio-economic groups, and other relevant sub-population characteristics. Other work has found that in low income settings a number of social determinants of health interact in the presentation of NCDs – including water, education, transportation and opportunities for physical activity [57]. The next step in contextualising the differences found across areas compared in this study, would be to assess a wider range of determinants and their intersections - using both qualitative and quantitative methods - to understand why some Supervision Areas fared better. Indeed, as screening rates increase, it is possible that NCD prevalence itself may decline due to a denominator effect (all the more so because the eligibility criterion in India includes those aged 30 and over, while global recommendations call for screening in those aged 40 and over). Equity-oriented oversight of screening, diagnosis, treatment and follow up in population subgroups should be a key feature of monitoring CPHC rollout going forward.

### Strengths and limitations

This LQAS study is among the first of its kind – assessing coverage of NCD services in urban and rural contexts across two Indian states. It takes a step further by trying to compare coverage by sex and poverty status. The strengths of LQAS, as noted previously [34], include the relatively small sample size required, the dichotomous program focussed measurement (achieved program target or not), and the possibility of fieldwork being carried out by program staff (without needing researchers). Adding to this, our study demonstrated the feasibility of using LQAS for measuring inequality in program coverage, especially in resource limited settings where larger sample sized sampling methodologies are not possible. Notwithstanding this, we were not able to achieve samples of BPL populations in many instances, given geographic housing segregation in both rural and urban settings. This is a constraint in applying LQAS for measuring inequality, where those inequalities might not be spread equally spatially (example, BPL populations may not live in areas where APL populations do) and are not visible using frequentist approaches. More methodological work on the domain is required to explore and understand the issue.

All the priority indicators in our study were assessed based on self-report; they are therefore subject to social desirability bias and other sources of error. Prior research has found that self-reported diagnoses and standardized criteria for assessment of NCDs differ markedly [58]. That said, self-reported data is routinely relied on for assessment of service utilisation and coverage, including by India’s National Sample Survey Organisation [59]. Triangulation of these findings with clinical diagnosis may offer further insights, alongside methods that triangulate with administrative data.

This study was a one-off exercise to test feasibility. Repeated LQAS embedded as a component of program monitoring would allow us to get an understanding of temporal trends as well as allow analysis of programmatic facilitators and barriers by sharing learnings across different Supervision Areas. This is particularly important since the NCD programme is in its early phases and must be administered across widely varying contexts and circumstances. Additionally, this report only presents analyses using the sampling groups. Further analyses are proposed that will look at the entire sample to analyse key outcomes. Finally, this study looks at inequalities; as aforementioned, we require gather additional (types of) data to determine whether these inequalities are avoidable, unfair, and unjust to be able to indicate whether they are inequities.

## Conclusion

In conclusion, this study demonstrated that while recall and penetrance of frontline workers is high, blood pressure and blood sugar screening coverage still fall far short of the established coverage thresholds in both rural and urban settings of India. This is partly to be expected, given that we are in early stages of the programme. Other than this, the project has demonstrated the feasibility of carrying out LQAS for NCD service coverage, while also helping to identify entry-points for service coverage improvement – which we have found to vary from location to location. Future research must seek to explore - and programme strategies expand - access pathways by looking at gender and poverty status, as well as other important social determinants.

## Supplementary Information

**Additional file 1.**

## Data Availability

The datasets used and/or analysed during the current study are all present in the tables in raw form. Additional information is available from the corresponding author on reasonable request.

## Abbreviations

ANM: Auxiliary Nurse/Midwife
CVD: Cardiovascular Disease
CPHC: Comprehensive Primary Health Care
LMIC: Low and Middle-Income Country
NCD: Non-Communicable Disease
PHC: Primary Health Centre
RMNCAH: Reproductive, Maternal, Newborn, Child and Adolescent Health
UHC: Universal Health Coverage

## Footnotes

1 We used APL and BPL as a proxy for socio-economic status as a number of health and social welfare services are made available preferentially to those Below the Poverty Line in India, as has been done in other studies [48–51]. Poverty lines, though contentious, are used to target development and welfare schemes in many countries to reduce class/income-related inequalities in access to public goods.
