# Supplementary material for: Assessing cardiovascular disease risk factor screening inequalities in India using Lot Quality Assurance Sampling
Source: BMC Health Serv Res. 2020 Nov 25;20:1077. doi: 10.1186/s12913-020-05914-y (PMC7687829; doi:10.1186/s12913-020-05914-y)
Supplement: Supplementary file 1 — Additional file 1. [file 12913_2020_5914_MOESM1_ESM.pdf]

LANDING SCREEN

Lot Quality Assurance Sampling Questionnnaire for

Assessing coverage, inequalities, and frontline provider workflows for hypertension and diabetes screening, treatment, and follow-up in two Indian states

Enter Password here to proceed in English

लोट क्वालिटी अशरन्स साम्प्लिंग प्रश्नावली :

दो भारतीय राज्यों में ग़ैर संक्रामिक बीमारियों की जाँच और देखभाल की व्याप्ति, असमानता, और समुदायिक स्वास्थ्य कार्यकर्ता की गठिविधियों के ऊपर शोध

हिंदी में आगे बढ़ने के लिए, यहाँ पैस्वर्ड भरिए

**SCREEN 1**							
**A. BACKGROUND**							
1	Date (day/month/year):						
2	Location			Delhi – Central		1	
				Delhi – South		2	
				UP – Shravasti- Sirsia		3	
				UP – Jhansi -Bhojla		4	
3	SHC/ANM Code /						
4	Name of Surveyor ________________			5	Supervisor ID		
6	Sampling group			7	Asha Name		
	Female	1					
	Male	2					
	APL	3					
	BPL	4					
8	Have you shared a participant information sheet?	Yes	1	9	Have you completed the written informed consent sheet?	Yes	1
		No	0 - STOP POPUP - “You cannot proceed without sharing the sheet”			No	0- STOP POPUP - “you cannot proceed without having proof of written informed consent”

The George Institute for Global Health India

NHSRC

SCREEN 2				
B. Individual Information				
Sl.No	Question and Filters	Code Response Categories		Skip To
1	Gender	Female	1	
		Male	2	
		Transgender	3	
2	What is your age	Number of years		
3	What is your level of education?	Not literate (cannot read and write)	0	
		Primary or below	1	
		Secondary or below	2	
		Secondary and higher	3	
4	What religion do you belong to?	Hindu	1	If 88, activate short text field
		Muslim	2	
		Christian	3	
		Other, please specify	88	

SCREEN 3				
**B. Individual Information Continued**				
5	What caste do you belong to?	General	1	
		Other Backward Class	2	
		Schedule Caste	3	
		Schedule Tribe	4	
		Don't know/Won't say	99	
6	Are you married?	No	0	
		Married	1	
		Separated/Divorced	2	
7	Do you have a government-issued BPL card? /	No card	0	**If 88, activate short text field**
		(VERIFY) Below Poverty Line (BPL)	1	
		Are Below Poverty Line but no card	2	
		Other, explain	88	
8	What is your profession? What kind of work do you do?	Unemployed	0	**If 88, activate short text field**
		Agriculture– self - employed	1	
		Agriculture – labor	2	
		Non-agriculture labor/casual labor	3	
		Non-agricultural - Self-employed	4	
		Service/salaried	5	
		Home maker	6	
		Other, explain	88	

SCREEN 4				
**C. Community Based Assessment Checklist (CBAC)**				
9	Do you smoke or consume smokeless products like gutka or khaini?/	Never		0
		Used to consume in the past/sometimes now		1
		Daily		2
10	Do you consume alcohol daily?	No		0
		Yes		1
11	May I please have your waist measurement? *Confirm using inch tape (only men should measure men and women should measure women)*	Female	Male	
		<80 cm	<90 cm	0
		80-90 cm	90-100 cm	1
		>90 cm	>100 cm	2
		Permission not given		99
12	You described your work earlier. In a given week, would you say you spend 2.5 hours or more moving around and doing physical activity - like walking a lot or lifting or carrying things**?**	No		0
		Yes		1
13	Does any family member (parents or siblings) have high blood pressure, diabetes, or heart disease?	No		0
		Yes		1

SCREEN 5				
D. Interaction with ASHA/ANM & Screening				
14	Do you know the name of the ASHA working in your area?	No	0	**Have a7 appear for interviewer here – allow them to check if same**
		Yes – same as A7 (is correct)	1	
		Yes – but name is different from A7 (is incorrect)	99	
15	Has this ASHA visited your area in the past year? / (*Mark all that apply.)*	No	0	
		Yes	1	
		Don't know	99	
16	Has anyone from the nearby health facility (or an ASHA) come to speak to members of your household over the age of 30 in your family in the past year?	No	0	**If 0 or 99, skip to Screen 7**
		Yes	1	
		Don't know	99	

SCREEN 6					
E. Interaction with ASHA/ANM & Screening Continued I					
17	What information has this person gathered from you? /	a. Name and details of family members	**No**	0	
			**Yes**	1	
		b. Information about pregnancy	**No**	0	
			**Yes**	1	
		c. Information about whether I use tobacco	**No**	0	
			**Yes**	1	
		d. Information about whether I use alcohol	**No**	0	
			**Yes**	1	
		e. My waist circumference	**No**	0	
			**Yes**	1	
		f. Information about my family members having high blood pressure, diabetes, or heart disease	**No**	0	
			**Yes**	1	

SCREEN 7					
E. Interaction with ASHA/ANM & Screening Continued 2					
18	Did the ASHA give you any of the following information?				If 88, activate short text field
		a. What risk I have of getting chronic diseases	No	0	
			Yes	1	
		b. What days there is screening for NCDs	No	0	
			Yes	1	
		c. I should not smoke or chew tobacco, gutkha, khaini, etc.	No	0	
			Yes	1	
		d. I should not drink alcohol	No	0	
			Yes	1	
		e. I should reduce/maintain a healthy weight	No	0	
			Yes	1	
		f. I should exercise	No	0	
			Yes	1	
		g. I have risk of getting NCDs because of family history	No	0	
			Yes	1	
		h. There is a "fixed day" or "screening day" where I can get tested for NCDs	No	0	
			Yes	1	
		i. I should go to a PHC and get my pressure or sugar checked	No	0	
			Yes	1	
		j. I should go to a hospital and get admitted	No	0	
			Yes	1	
		k. I should see a private doctor	No	0	
			Yes	1	
		l. Gave me a card (verify that they have a family health card)	No	0	
			Yes	1	
		Other, explain		88	

SCREEN 8				
F. NCD screening, testing and follow-up				
19	When have you had your blood pressure checked?	Never	0	If 0 skip to Screen 10
		Within the past year	1	
		More than a year ago	2	

SCREEN 10				
F. NCD screening, testing and follow-up Continued 1a				
20	Where/how did your blood pressure screening happen?	Got tested at a VHND	1	**If 88, activate short text field**
		Attended an NCD screening day	2	
		Went to a PHC to get pressure checked	3	
		Went to see a private doctor	4	
		Had an emergency and found out during careseeking for that	5	
		Other, explain	88	

SCREEN 10				
**F. NCD screening, testing and follow-up Continued 1b**				
21	When have you had your blood sugar checked?	Never	0	If 0, go to screen 16
		Within the past year	1	
		More than a year ago	2	

SCREEN 11				
F. NCD screening, testing and follow-up Continued 2a				
22	Where/how did your blood sugar screening happen?	Got tested at VHND	1	
		Attended an NCD screening day	2	
		Went to a PHC to get sugar checked	3	
		Went to see a private doctor	4	
		Had an emergency and found out during careseeking for that	5	
		Other, explain	88	
23	Do you have high blood pressure/hypertension or diabetes?	Have neither	0	**If 88, activate short text field.**<br><br>**If 0 or 88 go to screen 16**
		Have diabetes only	1	
		Have hypertension only	2	
		Have both	3	
		Don't know	99	

SCREEN 12					
F. Confirmation, Treatment, Follow-up					
24	How did you find out about your most recent hypertension and/or diabetes diagnosis?	Attended an NCD screening day		1	**If 88, activate short text field.**
		Went to a PHC to get pressure or sugar checked		2	
		Went to see a private doctor		3	
		Had an emergency and found out during careseeking for that		4	
		Other, explain		88	
25	Which of the following happened during, immediately before or after your most recent diagnosis?	a. Got blood pressure checked	No	0	
			Yes	1	
		b. Got blood sugar checked	No	0	
			Yes	1	
		c. Received written diagnosis of hypertension- VERIFY that doctor note says “htn” or hypertension	No	0	
			Yes	1	
		**d.** Received written diagnosis of diabetes- VERIFY that doctor note says “DM” or diabetes mellitus	No	0	
			Yes	1	
		e. Received prescription for medication –	No	0	
			Yes	1	
		f. Received medication	No	0	
			Yes	1	
		g. Got referred to another facility	No	0	
			Yes	1	
		h. Got referred for more tests	No	0	
			Yes	1	

SCREEN 13				
**F. Confirmation, Treatment, Follow-up Continued 1**				
26	Was someone there to help you when you made your trip?	Nobody	0	
		ASHA	1	
		ANM	2	
		ASHA and ANM	3	
		Someone else (relative, etc.)	4	
27	Are you currently on diabetes or hypertension medication?	No	0	**If 0 go to screen 16**
		Yes	1	

SCREEN 14					
F. Confirmation, Treatment, Follow-up Continued 2					
28	How long have you been on medication?	Less than a month		1	**If 1 or 2, go to Screen 16**
		1-3 months		2	
		3 months or more		3	
		More than a year		4	
29	Have you had to stop any of your medication in the past three months?	a. Hypertension related	No	0	
			Yes	1	
		b. Diabetes related	No	0	
			Yes	1	
		c. Both	No	0	
			Yes	1	

SCREEN 15					
**F. Confirmation, Treatment, Follow-up Continued 3**					
30	Can we please see the results of your most recent blood sugar and/or blood pressure test?	a. Hypertension is under control– VERIFY LAST 3 BP MEASUREMENTS BELOW 140/90	No	0	
			Yes	1	
			Not available	99	
		b. Diabetes is under control- VERIFY HBA1C BELOW 8	No	0	
			Yes	1	
			Not available	99	
31	Have you had to spend any of your own money on any of these health related activities in the past three months?<br>*Tick 0 or enter emount*	a. Travel	No	0	
			Yes *Enter amount*		
		b. Medical Consultation (from Public)	No	0	
			Yes *Enter amount*		
		c. Medical Consultation (from Private)	No	0	
			Yes *Enter amount*		
		d. Diagnostic Tests (from Public)	No	0	
			Yes *Enter amount*		
		e. Diagostic Tests (from Private)	No	0	
			Yes *Enter amount*		
		f. Medicines (from Public)	No	0	
			Yes *Enter amount*		
		g. Medicines (from Private)	No	0	
			Yes *Enter amount*		
		h. Hospitalisation	No	0	
			Yes *Enter amount*		
		i. Other, explain	No	0	
			Yes *Enter amount*		

SCREEN 16
Thank you for participating. All the best to you for your health!
